# Polyacrylonitrile Nanofiber Membrane Modified with Ag/GO Composite for Water Purification System

**DOI:** 10.3390/polym12112441

**Published:** 2020-10-22

**Authors:** Wongi Jang, Jaehan Yun, Yejun Park, In Kee Park, Hongsik Byun, Chang Hyun Lee

**Affiliations:** 1 Department of Energy Engineering, Dankook University, Cheonan 31116, Korea; wjang@dankook.ac.kr (W.J.); kddyejun@gmail.com (Y.P.); inkee0149@gmail.com (I.K.P.); 2 Department of Chemistry, Illinois State University, Normal, IL 61790-4160, USA; jyun12@ilstu.edu; 3 Department of Chemical Engineering, Keimyung University, Daegu 42601, Korea

**Keywords:** silver nanoparticle, graphene oxide, nanocomposite, polymeric nanofiber, antibacterial, water treatment

## Abstract

Silver nanoparticle-modified graphene oxide (Ag/GO) was reliably prepared by using sodium borohydride (NaBH_4_) in the presence of citric acid capping agent via a simple wet chemistry method. This rapidly formed Ag/GO composite exhibited good dispersity in a solution containing hydrophilic polyacrylonitrile (PAN). Subsequent electrospinning of this precursor solution resulted in the successful formation of nanofibers without any notable defects. The Ag/GO-incorporated PAN nanofibers showed thinner fiber strands (544 ± 82 nm) compared to those of GO-PAN (688 ± 177 nm) and bare-PAN (656 ± 59 nm). Subsequent thermal treatment of nanofibers resulted in the preparation of thin membranes to possess the desired pore property and outstanding wettability. The Ag/GO-PAN nanofiber membrane also showed 30% higher water flux value (390 LMH) than that of bare-PAN (300 LMH) for possible microfiltration (MF) application. In addition, the resulting Ag/GO-PAN nanofiber membrane exhibited antibacterial activity against *Escherichia coli* (Gram-negative) and *Staphylococcus aureus* (Gram-positive). Furthermore, this composite membrane exhibited outstanding anti-fouling property compared to the GO-PAN nanofiber membrane in the wastewater treatment. Therefore, the simple modification strategy allows for the effective formation of Ag/GO composite as a filler that can be reliably incorporated into polymer nanofiber membranes to possess improved overall properties for wastewater treatment applications.

## 1. Introduction

Since the development of electrospun nanofibers, their potential applications have been expanded into various areas of research, such as biomedicine, energy, and environmental science [1,2,3,4,5,6,7]. Specifically, the electrospinning method, as a unique approach to prepare polymer nanofibers, has received great attention by virtue of its easy scale-up and high production rate with controlled structure [7,8,9]. A membrane designed from the resulting electrospun nanofibers can exhibit various features, including large specific surface area, high porosity, tunable pore size, and high permeability, which can ideally serve as a promising candidate as membrane-based filters for water treatment applications [10,11,12]. To obtain desired properties (e.g., pore size, porosity, pore distribution, etc.) for water filtration, diverse electrospinning conditions including process parameters (e.g., applied voltage, flow rate, tip-to collector distance) and environmental parameters (humidity and temperature) should be properly optimized via trial and error [12,13]. The numerous composite nanofiber membranes with nanomaterials have been designed upon loading diverse nanoscale fillers to improve their properties [14,15,16,17]. Although the use of fillers in electrospinning polymer can significantly provide enhanced functionality, however, it is often required for extensive optimization processes to avoid disturbing their original structures. Thus, the proper selection of fillers that can improve the functionality of electrospun nanofibers is still an ongoing challenge [18,19].

The water purification membrane is often needed for diverse functionalities to effectively interact with heavy metal ions and minimal antifouling along with improved physicochemical properties [20,21]. In particular, the current wastewater treatments are typically required for the combination of the membrane-based filtration and disinfection process (e.g., UV, ozone, chlorination, etc.) to remove pathogenic microorganisms and common foultants (e.g., sludge particles, colloids, proteins, oils, etc.) [22,23,24]. The deposition of these foultants in the membrane easily lead to a gradual decline of permeability during the wastewater treatment [25]. Recently, many advanced approaches have been studied for the reduction of these fouling issues but the most common processes are excess aeration and frequent chemical cleaning, which result in an increase of operating costs and energy demand [25]. Thus, current research in the development of membrane materials is mainly focused on overcoming these problems [26,27,28].

Graphene oxide (GO), one of the 2-D nanostructured fillers, is of particle interest because of its promising physicochemical property and easy modification to introduce functionality into polymer-based membranes even with the presence of very small amount [20,29,30]. Numerous hydrophilic functional groups, including edge and side chains of GO, can greatly improve the wettability and permeability of water during filtration. In addition, these hydrophilic oxygen groups can act as reactive oxygen species (ROS) to exhibit anti-bacterial property [31,32]. However, several researchers reported that the anti-bacterial impact of GO was somewhat limited due to size distribution and dispersion purity of GO [20,31,33]. Recently, silver nanoparticle/graphene oxide (Ag/GO) composites are considered as a promising material to overcome this limitation without any treatment [33]. In particular, the high specific surface area and oxygen-driven functional groups can readily allow for the immobilization of Ag nanoparticle via electrostatic interactions [33,34]. However, once silver nanoparticles are entrapped onto the GO sheets, their possible agglomeration can significantly reduce their dispersity in both water and organic solvents. For example, a reducing agent used for formation of Ag nanoparticles from Ag^+^ ions can induce the formation of reduced GO (rGO), which could result in an unexpected decrease of dispersity in a solvent when preparing for a precursor solution. Comparing with GO, the incorporation of relatively hydrophobic rGO into polymer matric can lead to a significant problem associated with the wettability and permeability of the final membrane [35]. Such immiscibility between rGO and polymer can lead to the formation of undesired defects, associated with a weak physicochemical property, in the overall morphology of the membrane. As such, with respect to membrane performance, current studies are required for further understanding and investigating the effective incorporation of fillers into polymeric membranes.

In this work, exfoliated GO was initially modified with silver nanoparticles via a one-pot synthesis method. This simple synthesis allowed for the rapid formation of Ag nanoparticles and readily integrated onto the GO surface. The resulting Ag/GO nanosheets were mixed in the electrospun precursor solution containing PAN powder. Subsequent electrospinning was then reliably accomplished to prepare composite membranes which were comprehensively evaluated to understand how the loading of different GO-based fillers into nanofiber membranes impacts membrane performance (e.g., wettability, permeability, antifouling efficiency, etc.).

## 2. Experimental

### 2.1. Materials

Graphite flake (SP-1, Bay Carbon Inc., Bay City, MI, USA), sulfuric acid (H_2_SO_4_, ≥98%, Duksan Pure Chemical Co., Gyeonggi-do, Korea), potassium permanganate (KMnO_4_, ≥99.9%, Sigma-Aldrich, St. Louis, MO, USA), hydrogen peroxide (H_2_O_2_, 35%, Samchun Co., Seoul, Korea), and hydrochloric acid (HCl, 1 N, Duksan Pure Chemical Co., Gyeonggi-do, Korea) were used for the synthesis of graphene oxide (GO). Silver nitrate (AgNO_3_, ≥99.9%, MW = 169.87 g/mol, Sigma-Aldrich, St. Louis, MO, USA), sodium citrate (C_6_H_5_Na_3_O_7_∙2H_2_O, ≥98.0%, MW = 294.09 g/mol), and sodium borohydrate (NaBH_4_, ≥99.0%, ACROSS Organics, Geel, Belgium) were used for the synthesis of Ag and Ag/GO nanoparticles. Polyacrylonitrile (PAN, MW = 150,000 Da, Sigma-Aldrich, St. Louis, MO, USA) and N,N-dimethyl formamide (DMF, >99.0%, Duksan Pure Chemical Co. Ltd., Gyeonggi-do, Korea) were used for the preparation of electrospun precursor solution. Nanopure water was obtained using a Millipore system (~18 MΩ) and all chemicals were used as received without further purification.

### 2.2. Synthesis of Ag/GO Nanocomposite

Graphene oxide (GO) was initially prepared by slightly modifying the Hummers method (oxidation and exfoliation of graphite) [2,36,37]. This GO solution was filtered with a 0.45 µm of PVdF filter (Millipore Co., Ltd., Burlington, MA, USA) using a dead-end-cell device under 2 bar pressure and then completely dried in an oven at 60 °C. The thin layer of GO sheets (0.02 g) was in 100 mL nanopure water and then completely dispersed via sonication for 2 h prior to use. Ag/GO nanoparticles were synthesized by slightly modifying the chemical reduction process [38]. An aliquot (1.0 mL) of 2 wt% AgNO_3_ solution and 1 mL of citric acid (CA, 1 wt%) were mixed in 100 mL of GO solution prepared at 0.2 mg/mL density under vigorously stirring for 30 min. Then, 2 mL of NaBH_4_ solution (100 mmol, 0.019 g NaBH_4_ in 5 mL H_2_O) was quickly added in this mixture to rapidly form Ag nanoparticles on the surface of GO. The resulting solution was filtered by a vacuum filtration system with a 0.45-µm pore diameter of PVdF filter (HVLP09050, Durapore membrane filter, Millipore Co., Ltd., Burlington, MA, USA). The Ag/GO nanosheets were then obtained after completely dried them in an oven at 60 °C for 24 h.

### 2.3. Fabrication of Ag/GO-PAN Nanofiber Membrane

The bare PAN, PAN/GO, and Ag/GO-PAN nanofiber membrane were prepared by our developed approach [29,30,39]. Initially, the synthesized GO (0.02 g) and Ag/GO nanosheets (0.02 g) were completely dispersed in a DMF solvent (10 mL) by sonicating for 2 h at rt. Then, 1.0 g of PAN powder was fully dissolved in this dispersion solution under vigorously stirring overnight. This precursor solution was transferred to 5 mL of a plastic syringe equipped with a 23 gauge needle, which was then electrospun under the following condition: applied voltage of 15 kV, the flow rate of 0.8 mL/h, tip to collector distance of 15 cm, drum speed of 200 rpm. The collected nanofiber mat was hot-pressure-treated under 40 °C and 6000 psi after folding the layers to improve the physical property and to control the pore property for water purification. The resulting nanofiber membrane was sequentially rinsed with nanopure water and ethanol to remove the residuals, and then completely dried prior to use.

### 2.4. Characterization

To optimize the synthesis conditions for Ag particles and Ag/GO composites, the ultraviolet-visible (UV-vis) spectroscopy analysis was performed by a UV-1650PC (Shimadzu Europe, Manchester, UK) under a range from 200 to 700 nm. The overall morphology of prepared samples was characterized by a scanning transmission electron microscope (STEM, Hd-2300, Hitach, Tokyo, Japan) and scanning electron microscope (SEM, S-4800, Hitachi, Tokyo, Japan). STEM samples were prepared by completely drying 1-2 drops of GO and Ag/GO solution on a TEM grid (400 mesh carbon) under infrared light. The STEM measurement was then performed at the acceleration voltage of 200 KeV. SEM samples were prepared by completely drying in an oven at 60 °C overnight, and a thin layer of osmium (Os) was coated onto the surface of the sample for 10 sec under 50 torr by a vacuum sputter. Raman spectroscopy (a bench-top ProRaman-L Analyzer, Optronics, CA, USA) and Fourier transform infrared spectroscopy (FTIR, FT/IR-620V, JASCO, Tokyo, Japan) were used for the characterization of chemical structure of prepared nanoparticles (NPs) and nanofiber membranes. The Raman data were collected using 10 mW of 785 nm laser with 10 s acquisition time. For the FT-IR analysis of the NPs, the samples were prepared by mixing with KBr disc with 1/50 ratio. The pore properties of nanofiber membranes were examined by a capillary flow porometer (Porolux 1000, BT-FT GmbH, Berlin, Germany) using N_2_ gas. The sample was fully soaked in a Porewick standard solution with a 16.0 dynes/cm surface tension for 30 min, and the measurement was carried out under wet and dry method. The porosity of nanofiber membranes (5.0 cm × 5.0 cm) was measured by comparing the dry and wet weights of the membrane after soaking them in n-butanol for 2 h. The porosity was then calculated by the following Equation (1):

(1) $$P\left(\%\right)=\;\frac{W_{w}\;-\;W_{d}}{\rho_{b}\;\times \;V_{d}}$$

where *W_w_* is the weight of the wet membrane, *W_d_* is the weight of the dry membrane, *ρ_b_* is the density of n-butanol, and *V_d_* is the volume of the dry membrane. The thickness of the nanofiber membrane was measured by a digital Vernier caliper (ABS Digimatic Thickness Gauge, Mitutoyo Corp., Kawasaki, Japan). The wettability change of the nanofiber membrane was monitored by a contact angle analyzer (Phoenix 300, SEO Inc., Gyeonggi-do, Korea) using a water droplet.

### 2.5. Antimicrobial Activity of PAN/Ag/GO Composite Nanofiber Membrane

To demonstrate the antibacterial activity of the prepared nanofiber membranes, two different bacterial strains (Gram-negative; *E. coli.* (−) and Gram-positive; *S. aureus* (+)) were employed in this study. The bacterial strains were grown until the absorption peak ranging from 0.132 to 0.18 at 600 nm, which indicates about 108 Colony-forming unit (CFU). 150 µL of the grown bacterial suspension was inoculated individually in 10 mL of nutrient broth (NB) by the disc diffusion method (Kirby-Bauer method). The sample (6 mm of a disc shape) was placed on the top and the zone of inhibition was recorded after incubation them at 37 °C for 24 h in an oven.

### 2.6. Water flux and Antifouling Test of PAN/Ag/GO Composite Nanofiber Membrane

**Scheme 1:**
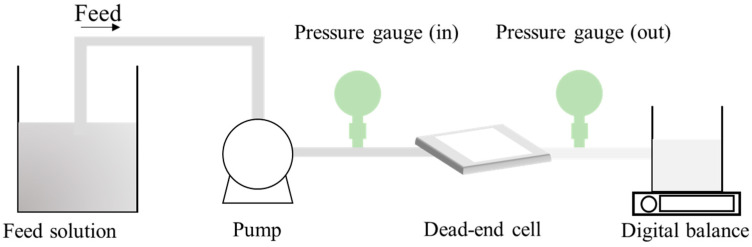
The schematic diagram of water treatment process.

The membrane performance and antifouling test were carried out using a fixed dead-end device. The pure water flux test was carried out using a 0.00310 m^2^ membrane cell and deionized water at 50 kPa. The weight change was measured as a function of time and calculated by the following Equation (2).

(2) $$J_{i}=\frac{Q}{A\Delta t}$$

where *J_i_* is water flux (L/m^2^·h), *Q* is water permeate volume (L), *A* is an effective area of the sample (m^2^), and *t* is permeation time (h). The trans membrane pressure (TMP) of the membrane was measured under 100 LMH of constant permeate with swine wastewater, which was obtained in a wastewater treatment plant in Korea. The TMP change was monitored in real-time by a directly connected pressure gauge. Scheme 1 shows the overall water treatment process.

## 3. Results and Discussion

### 3.1. Structure and Morphology of Synthesized Nanoparticles and Nanofiber Membranes

**Figure 1:**
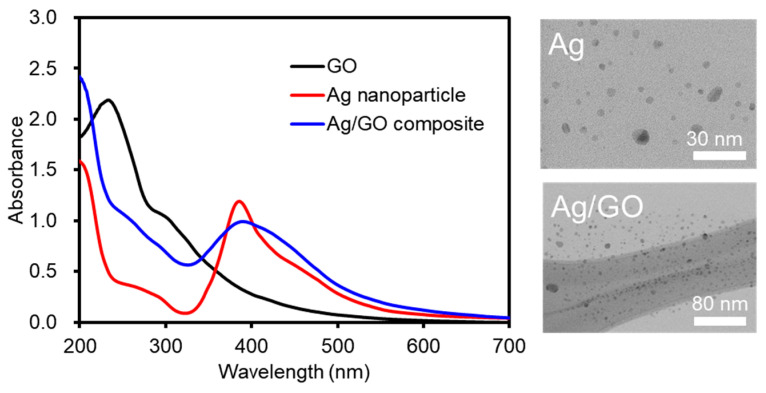
UV-vis spectra of synthesized nanoparticles and their presentative STEM images.

Figure 1 shows the optical property of synthesized GO, Ag nanoparticle (AgNP), and Ag/GO composite. The absorption spectra of the AgNP colloidal solution indicated the conventional peak at ~390 nm associated with the formation of small and spherical AgNPs [38,40]. The particle size of AgNPs was estimated to 4.7 ± 2.5 nm (Figure 1), which was well-matched with its plasmonic resonance peak. In addition, the use of the NaBH_4_ reducing agent allowed for the rapid formation of AgNPs (~5 min) at room temperature, and no significant change of the plasmonic band was observed as a function of time shown in Supplementary Figure S1. As such, the synthesis of the Ag/GO composite was carried out under the same reaction conditions. Comparing to the absorption peak of bare AgNPs, the Ag/GO composite had a relatively broad absorption band between 330 nm and 500 nm. This result was expected because of possibly localized agglomeration of randomly deposited AgNPs onto the GO surface whereas the agglomeration is not typically observed in small and monodispersed metal NPs in water [41]. In addition, the particle size of AgNPs (11.7 ± 7.1 nm) of the composite was unexpectedly increased up to 2.5 times compared to that of the bare AgNPs, leading to the change of the plasmonic bandwidth and position.

The synthesized GO possessed two different absorption peaks at 237 nm for π-π* transition mode of aromatic C–C bond and at 300 nm for n-π* transition mode of C=O bond, respectively [40,42]. Unlike the GO, such characteristic peaks were not observed for the Ag/GO composite in an absorption spectrum, possibly due to their chemical structure change during the synthesis process (e.g., GO to rGO) [43]. Nevertheless, the particle size was still relatively smaller, and the composite showed a better dispersion under our experimental conditions than those of the thermal-driven method (Supplementary Figure S2). It was anticipated that the relatively high dispersion characteristics of the prepared Ag/GO composites were contributed from the use of NaBH_4_. In other words, NaBH_4_ tends to have excellent reducing power for –C=O groups, but relatively low for –COOH and –OH species [43]. To understand the tendency of the reducing behavior of NaBH_4_, the prepared NPs were examined via FT-IR (Supplementary Figure S3). The Ag/GO exhibited several strong vibrational peaks at ~3300 cm^−1^ (–OH), 1620 cm^−1^ (–C=C), 1050 cm^−1^ (–C–O–C), but the relatively weak intensity of –C=O vibrational peak at 1710 cm^−1^. Therefore, the remaining –OH and/or –COOH group in the Ag/GO composite could enable their desire dispersity in an organic solvent.

**Figure 2:**
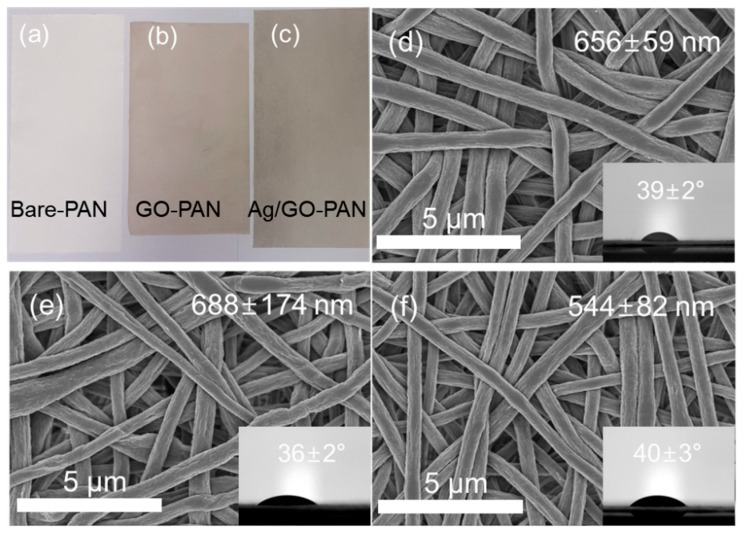
Digital photos (**a**–**c**), SEM images (**d**–**f**), and contact angle of nanofiber membrane: (**a**,**d**) bare-PAN, (**b**,**e**) GO-PAN, and (**c**,**f**) Ag/GO-PAN nanofiber membranes, where the top right value indicates the average diameter of nanofibers.

Figure 2 shows the digital photo and SEM images of bare-PAN, GO-PAN, and Ag/GO-PAN nanofiber membranes. The color of GO and Ag/GO modified nanofibers changed from white to uniformly dark gray across the membrane. The notable defects were not observed in both digital photos and SEM images, which implies that the hydrophilic nature of PAN could allow for the successful incorporation of the synthesized nanofillers into the nanofibers via electrospinning. However, the average diameter of individual nanofiber strands and its distribution showed distinctively different patterns upon the addition of GO and Ag/GO nanocomposite. The GO-incorporated nanofibers displayed thicker fiber strands with a broad distribution compared to that of bare-PAN nanofibers (656 ± 59 nm for bare-PAN and 688 ± 177 nm for GO-PAN, respectively), while the Ag/GO-incorporated nanofibers showed thinner fiber strands (544 ± 82 nm). In order to understand this structural feature, the viscosity of precursor solution was examined prior to electrospinning because this parameter is considered as an important factor to determine the final fiber diameter during electrospinning (e.g., the higher the viscosity, the smaller the fiber diameter) [44]. However, after the addition of GO and Ag/GO composites into the PAN solution, the viscosity slightly increased from 840 cp (bare PAN solution) to 890 cp and 875 cp (GO and Ag/GO-PAN solution, respectively). This result suggested that the miscibility between the nanofillers and polymer nanofibers can act as an important factor to determine the characteristics of final diameters. The increase in the fiber diameter of the GO-PAN nanofibers was anticipated due to the excellent miscibility (e.g, hydrophilicity) between the GO and PAN. This led to a formation of the embedded state of GO, implying that most GO was embedded within PAN nanofibers. Whereas, Ag/GO-PAN nanofibers showed a poor miscibility due to slightly different polarities which led to a decrease in fiber diameter and poor distribution of the nanofibers. The contact angle measurement was carried out to confirm the effect of fillers on the surface polarity of the final membrane. The wettability, permeability of the membrane were significantly reduced, which confirmed the importance for utilizing fillers in the water purification system [30]. The water contact angle of the nanofiber membrane slightly decreased from 39° to 36° upon the incorporation of GO, but the Ag/GO-PAN nanofiber membrane exhibited a slight increase of the angle from 39° to 40°. This result strongly supported that Ag/GO composite has more hydrophobic characters than GO due to their higher reduction of oxygen functional groups (e.g., –C=O, –COOH, –OH) during the synthesis process [45].

**Figure 3:**
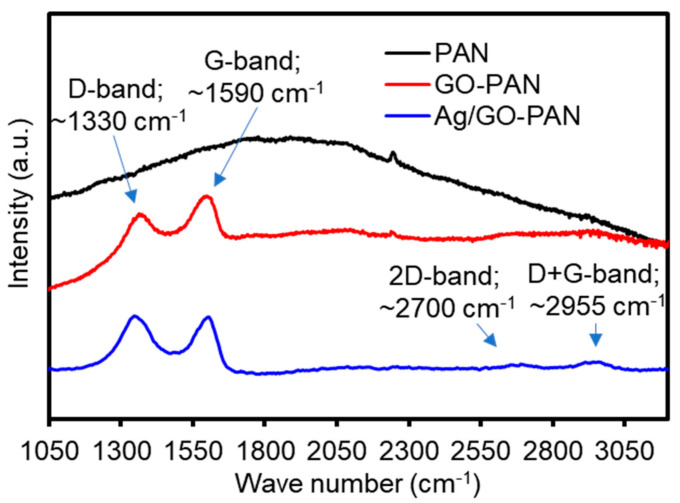
Raman spectra of nanofiber membranes.

Raman spectra of the prepared nanofiber membranes were examined to further understand the chemical structures of GO and Ag/GO composites and their incorporation into PAN nanofiber (Figure 3 and Supplementary Figure S4). The GO- and Ag/GO-PAN nanofiber membranes exhibited two strong peaks at 1330 cm^−1^ associated with D-band and 1590 cm^−1^ associated with G-band, respectively [37]. However, the bare-PAN nanofiber membrane did not show such distinctive peaks in all spectra. This result clearly supported the presence of GO and Ag/GO composite into the PAN polymer matrix. Herein, we noted the difference of D-band and G-band ratio between GO and Ag/GO composite to understand their polarity. As reported in previous literature, the G-band corresponds to the in-plane vibration of sp^2^ carbon domains and the D-band is associated with the structural defects and edges of GO, indicating the out of plane vibration for sp^3^ and sp carbon domains [46,47]. Thus, comparing the I_D_/I_G_ ratio of GO and Ag/GO composite, the miscibility between the PAN nanofibers and fillers synthesized under our experimental conditions can be predicted. As indicated in Supplementary Figure S4, the I_D_/I_G_ ratio of synthesized GO was estimated to ~1.07 and the ratio notably increased to ~1.47 after the incorporation ofAgNPs (Ag/GO), indicating the increase in structural defects caused by broken sp^2^ carbon domains [48]. This result implies that the NaBH_4_ can lead to an increase in the sp^3^ and sp carbon domains and decrease the oxygen functional groups in GO [45]. As such, this chemical structure change in GO can also lead to a decrease of its hydrophilic nature, which can affect increasing the reduction of miscibility with the hydrophilic PAN nanofiber.

### 3.2. Antimicrobial Activity and Water Treatment Characterization of Nanofiber Membranes

**Figure 4:**
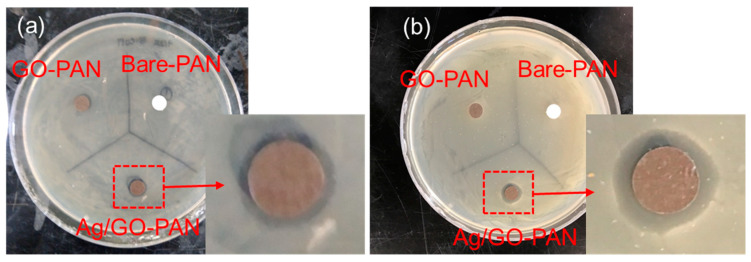
Antimicrobial activity of nanofiber membranes against (**a**) *Escherichia coli* (gram-negative) and (**b**) *Staphylococcus aureus* (gram-positive).

Figure 4 and Table 1 show the antibacterial activity of nanofiber membranes incorporated with GO and Ag/GO composite against Gram-negative (*E. coli*) and Gram-positive (*S. aureus*) bacteria. The Ag/GO-containing PAN nanofiber membrane exhibited an applicable antimicrobial activity against both Gram-negative and Gram-positive bacterial strains, which were compared to those of the GO-PAN nanofiber membrane. This result was expected that the GO fillers were not directly exposed to the bacterial strain since the hydrophilic nature led to the formation of an embedded state of GO that can readily occur due to their good miscibility. With this assumption, the control experiment was carried out with hydrophobic polymer (polyvinyl fluoride, PVdF) nanofibers containing GO and rGO for the Gram-positive bacteria because it only contained a single layer of peptidoglycan outside of cell membrane which led to a relatively quick comparison of antibacterial effect compared to the Gram-negative bacteria (Supplementary Figure S5). Interestingly, the GO-PVdF nanofiber membrane exhibited a good antibacterial activity, which was clearly shown from that of GO-PAN nanofibers. The results suggested that the presence of GO between the polymer and bacterial strain can prevent their adhesion and growth due to the electrostatic repulsion and chemical interaction of GO [20,31,32]. Therefore, the physicochemical nature of GO incorporated into polymer matric can be considered as an important factor to control the anti-biofouling activity of the final membrane for the water purification system. However, the GO-PVdF nanofibers were not considered in this study due to the poor reproducibility.

**Figure 5:**
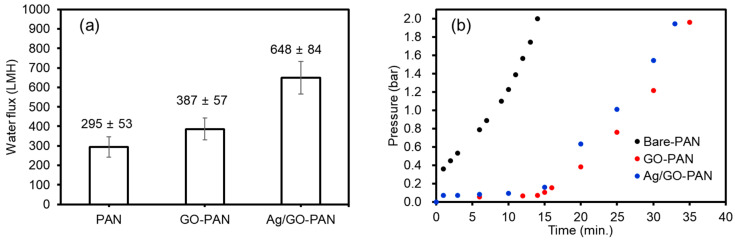
(**a**) Purewater permeability and (**b**) trans membrane pressure (TMP) of the nanofiber membrane. (Note; the TMP was tested with harsh wastewater).

To utilize the prepared nanofiber membrane in the water treatment system, the general characteristics of the membrane were initially examined by monitoring the pore size and porosity prior to the water permeability test. The bare-PAN possessed ~356 nm of average pore size and after the addition of GO and Ag/GO composite the pore size became ~385 nm for GO-PAN and ~243 nm for Ag/GO nanofiber membrane, which was associated with their fiber diameters (Figure 2, vide supra). Whereas, the porosity of the nanofiber membranes was estimated to be similar 48–52% within the experimental standard deviation. From this result, it was evident that all the membranes prepared were suitable for a MF grade membrane. The water permeability of the prepared nanofiber membranes was examined as a function of time at 50 kPa (Figure 5a) without any wetting processes (e.g., alcoholic solution treatment) because all the membranes possessed a superior wettability for the water source. As shown in Figure 5a, the bare-PAN membrane exhibited the lowest water permeability (~300 LMH), while the GO-PAN membrane showed over two times higher values (~650 LMH), possibly due to its improved hydrophilicity and bigger pore size. Despite its small pore size, Ag/GO-PAN showed slightly increased water permeability (~390 LMH) compared to that of bare-PAN which possibly due to its pore distribution and shape associated with the tortuosity of cross-section [15,39]. Therefore, the interconnected status of nanofillers into polymer matrix can highly influence the mass transport of water through pore channels by varying pore size, pore uniformity, and tortuosity of the cross-section. 

Additionally, the antifouling activity of the prepared nanofiber membranes was conducted with hash wastewater (+9999 NTU) at the constant flux of 100 LMH by monitoring the reaching time to 2 bar trans membrane pressure (Figure 5b and Supplementary Figure S6). In case of the bare-PAN nanofiber membrane, the TMP was rapidly increased with the inflow of wastewater, while the GO- and Ag/GO-containing nanofiber membranes did not change much during the first 15 min. This observation could be explained that more negatively charged GO and antibacterial Ag/GO composite prevented the accumulation of organic foultants on the surface and/or inside of the membrane [49,50]. However, they showed a similar tendency to that of the bare-PAN after 15 min operation. This was anticipated that the effects of the GO and Ag/GO composite significantly declined as the pressure of the inflow of wastewater increased. This result showed that the GO and Ag/GO-containing PAN nanofiber membranes exhibited an outstanding antifouling efficiency over the bare-PAN nanofiber membrane. Thus, the modification of polymer nanofiber membrane with a small amount of Ag/GO composite can allow for improving various properties of the composite membranes. Many variables should still be optimized for the development of nanofiber-based membranes for water purification system, so our future study will involve the characterization of selectivity of organic debris (e.g., protein) and/or inorganic species (e.g., heavy metal ions).

## 4. Conclusions

In this work, silver nanoparticle modified graphene oxide (Ag/GO) was reliably prepared via a one-pot synthesis method using sodium borohydride (NaBH_4_) in the presence of citric acid capping agents. The prepared NPs were characterized by STEM, UV-vis, FT-IR, Raman spectroscopy to examine their chemical structure, size, and shape, which served as fillers in the polymeric nanofiber membrane. This simple strategy allowed for the rapid formation of Ag nanoparticles and readily entrapped them on the GO surface. The resulting Ag/GO composite exhibited a good dispersity under our experimental conditions due to the presence of oxygen functional group. Subsequent electrospinning of a precursor solution with the hydrophilic polyacrylonitrile (PAN) and Ag/GO composite resulted in the successful formation of nanofibers without any notable defects. The resulting Ag/GO-PAN membrane was comprehensively evaluated by comparing with the bare-PAN and GO-PAN membrane in order to understand how the loading of different GO-based fillers impacts the overall properties of nanofiber membranes and their water purification performance. These results showed that the Ag/GO-PAN nanofiber membrane possessed thinner fiber strands (544 nm) and smaller pore size (243 nm) compared to that of Bare-PAN. This observation implied that the miscibility between GO-based nanofillers and polymer could play a key factor in controlling the fiber diameter and the pore size. In addition, the Ag/GO-PAN membrane exhibited greater antibacterial activity, 30% increasing water permeability, and anti-fouling efficiency than the bare-PAN nanofiber membrane. Overall, this simple modification strategy to effectively incorporate interesting fillers into nanofiber membranes could provide a guide to greatly improve the general properties of pristine nanofiber membrane when applying to a MF-based water purification system.

## Figures and Tables

**Table 1:** Inhibit zone diameter (mm) of prepared nanofiber membranes for *Escherichia coli* and *Staphylococcus aureus.*

Sample	*Escherichia coli*	*Staphylococcus aureus*
Bare-PAN	-	-
GO-PAN	-	-
Ag/GO-PAN	1.0 ± 0.2	2.2 ± 0.3

## Supplementary Materials

The following are available online at https://www.mdpi.com/2073-4360/12/11/2441/s1, Figure S1. UV-vis spectra of Ag nanoparticle colloidal solution synthesized by using NaBH4 reducing agent as function of time. Figure S2. STEM image of Ag/GO composite synthesized via thermal-driven process. Figure S3. FT-IR spectra of GO and Ag/GO composite. Figure S4. Raman spectra of prepared nanofiber membrane after baseline correction. Figure S5. Antimicrobial activity of PVdF nanofiber membrane loaded GO-based nanomaterials for gram-positive bacteria (Staphylococcus aureus). Figure S6. (a) Feed Wastewater and (b) dead-end-cell device for water permeability and antifouling characterization of nanofiber membranes.
