# Supplementary material for: Polyacrylonitrile Nanofiber Membrane Modified with Ag/GO Composite for Water Purification System
Source: Polymers (Basel). 2020 Oct 22;12(11):2441. doi: 10.3390/polym12112441 (PMC7690632; doi:10.3390/polym12112441)
Supplement: Supplementary file 1 [file polymers-12-02441-s001.pdf]

polymers

MDPI

Article

Polyacrylonitrile Nanofiber Membrane Modified with Ag/GO Composite for Water Purification System

Wongi Jang [1,†], Jaehan Yun [2,†], Yejun Park [1], In Kee Park [1], Hongsik Byun [3,*] and Chang Hyun Lee [1,*]

[1] Department of Energy Engineering, Dankook University, Cheonan 31116, Korea; wjang@dankook.ac.kr (W.J.); kddyejun@gmail.com (Y.P.); inkee0149@gmail.com (I.K.P.)
[2] Department of Chemistry, Illinois State University, Normal, IL 61790-4160, USA; jyun12@ilstu.edu
[3] Department of Chemical Engineering, Keimyung University, Daegu 42601, Korea
* Correspondence: hsbyun@kmu.ac.kr (H.B.); chlee@dankook.ac.kr (C.H.L.)
† These authors equally contributed to this work.

Received: 31 August 2020; Accepted: 19 October 2020; Published: date

Supplementary Material

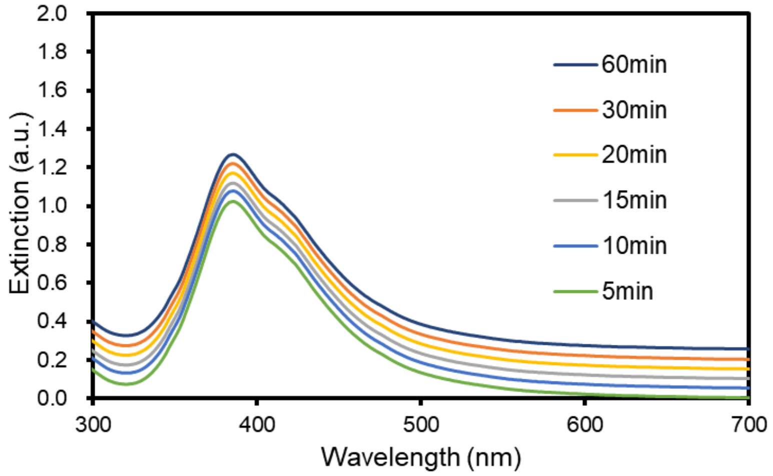

Figure S1. UV-vis spectra of Ag nanoparticle colloidal solution synthesized by using $NaBH_4$ reducing agent as function of time.

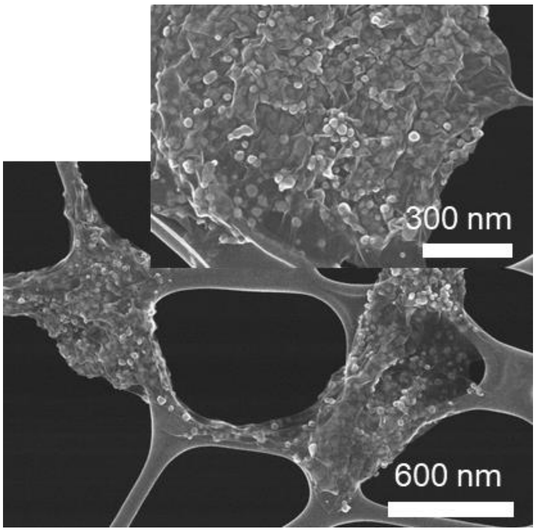

Figure S2. STEM image of Ag/GO composite synthesized via thermal-driven process.

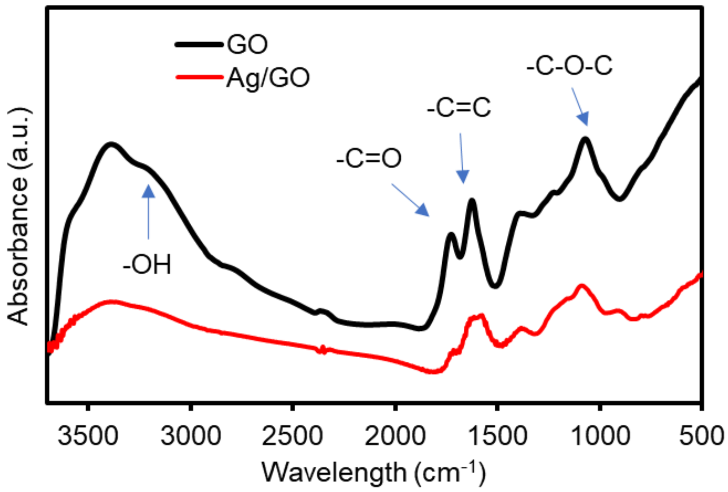

Figure S3. FT-IR spectra of GO and Ag/GO composite.

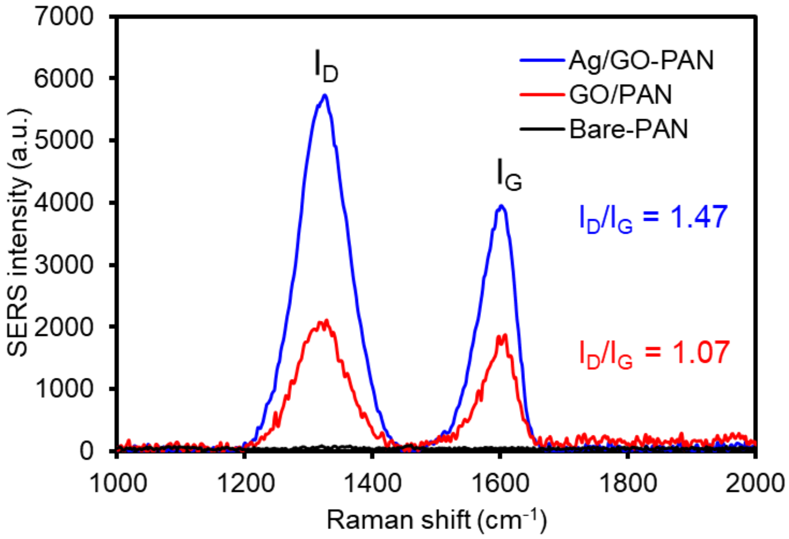

Figure S4. Raman spectra of prepared nanofiber membrane after baseline correction.

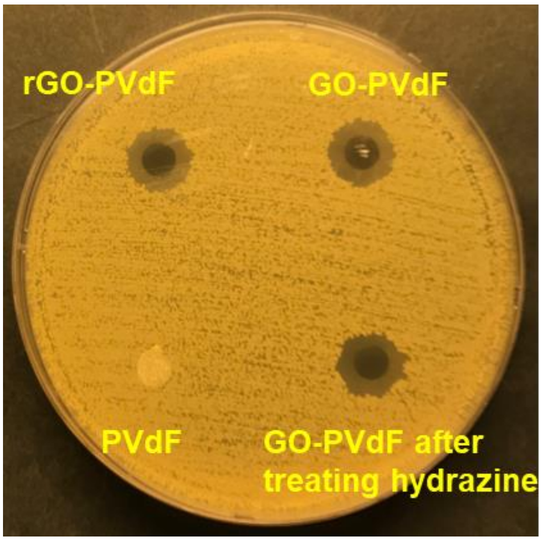

Figure S5. Antimicrobial activity of PVdF nanofiber membrane loaded GO-based nanomaterials for gram-positive bacteria (*Staphylococcus aureus*).

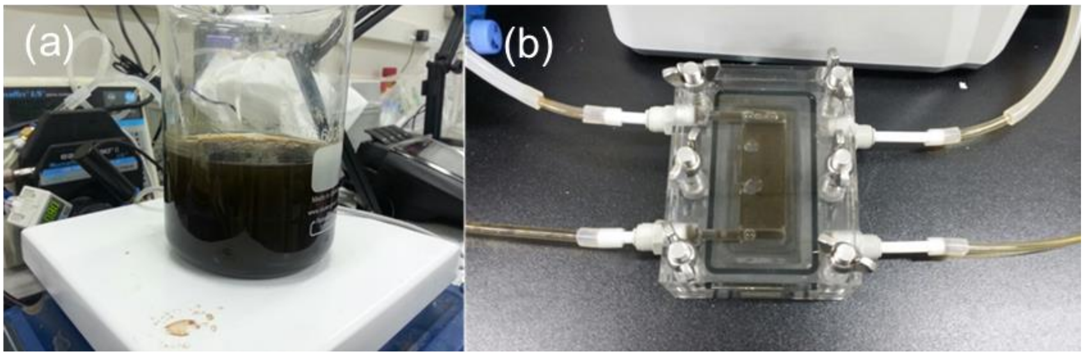

Figure S6. (a**)** Feed Wastewater and (b) dead-end-cell device for water permeability and antifouling characterization of nanofiber membranes.

© 2018 by the authors. Submitted for possible open access publication under the terms and conditions of the Creative Commons Attribution (CC BY) license (http://creativecommons.org/licenses/by/4.0/).
